# Ferumoxytol MR angiography for pre-TAVR assessment

**DOI:** 10.1186/1532-429X-18-S1-P338

**Published:** 2016-01-27

**Authors:** J Paul Finn, John Moriarty, Adam Plotnik, Takegawa Yoshida, Richard J Shemin, Olcay Aksoy, William M Suh

**Affiliations:** UCLA, Los Angeles, CA USA

## Background

CT angiography (CTA) is standard for assessment of arterial access anatomy prior to transcatheter aortic valve replacement (TAVR) procedures [1]. However, TAVR candidates are generally elderly with a higher prevalence of renal impairment than the general population and it is desirable to eliminate the use of iodinated contrast prior to catheterization because of the severe consequences of post-procedural renal failure [2]. Further, gadolinium based contrast agents may be problematic in renal impairment because of the perceived risk of NSF. We hypothesized that ferumoxytol may be a suitable alternative to CTA and Gd contrast enhanced MRA in these patients.

## Methods

Following informed consent and with approval from our IRB, we performed ferumoxytol enhanced MRA (FEMRA) for assessment of arterial access anatomy prior to TAVR in 20 patients (M/F = 15/5, mean age 84.5 yrs (+/- 8) with aortic stenosis and renal impairment (eGFR < 30 mls /m2.mn). FEMRA was performed at 3.0T in 15 patients and at 1.5T in 5, using a total dose of 4 mg /kg. Two patients had cardiac pacemakers. First pass and steady state FEMRA was performed in 16 patients and steady state imaging only in 4. The field of view extended from the neck to the proximal thighs in two overlapping stations. Images were post-processed with MIP and Volume Rendering.

## Results

**Figure 1:**
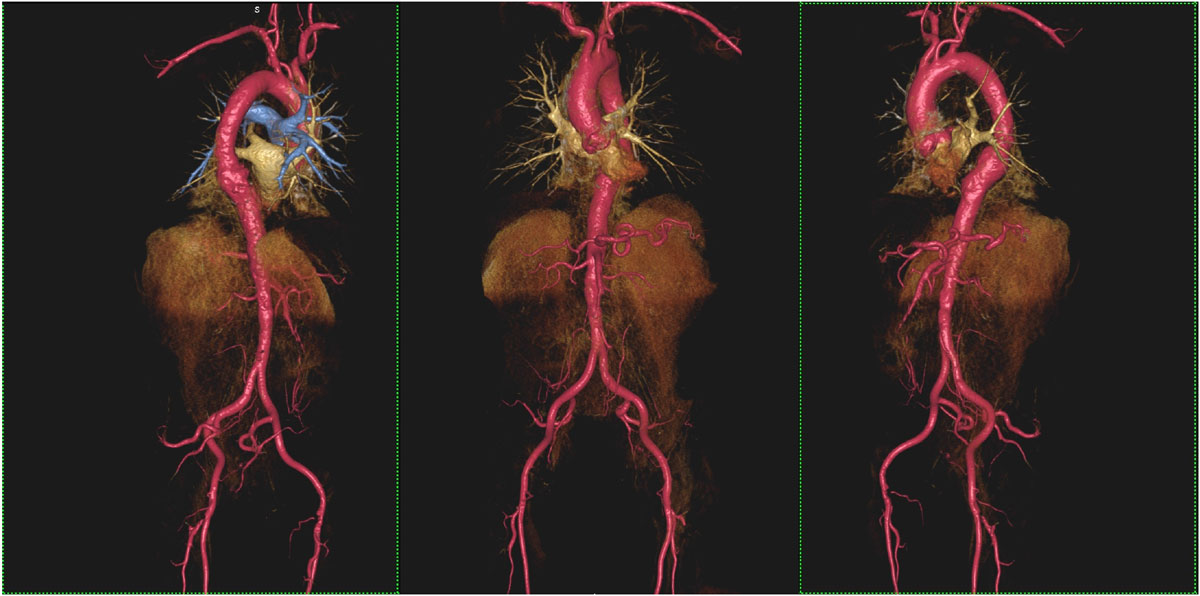
Figure 1

In all cases, FEMRA produced highly diagnostic studies, which formed the basis for confident TAVR planning. Figure 1 shows a volume rendered FEMRA study in an 87 year old male who subsequently underwent TAVR. All patients remained stable throughout the FEMRA procedures and there were no symptoms or significant changes in heart rate, blood pressure or blood oxygenation. Both first pass and steady state images were considered highly diagnostic. 12 patients had successful TAVR placement via a femoral approach with angiographic confirmation of FEMRA findings. 6 patients were not considered candidates for a femoral approach on the basis of FEMRA and had successful trans-apical placement. 2 patients are pending treatment.

## Conclusions

In the current study, FEMRA was diagnostic, practical and safe at both 3.0T and 1.5T and provided sufficient information for confident planning of the access route for TAVR. FEMRA holds promise as an alternative to CTA and Gd CEMRA in patients with renal impairment.
